# Association of inflammatory gene polymorphisms with ischemic stroke in a Chinese Han population

**DOI:** 10.1186/1742-2094-9-162

**Published:** 2012-07-06

**Authors:** Nan Zhao, Xin Liu, Yongqin Wang, Xiaoqiu Liu, Jiana Li, Litian Yu, Liyuan Ma, Shuyu Wang, Hongye Zhang, Lisheng Liu, Jingbo Zhao, Xingyu Wang

**Affiliations:** 1 Department of Epidemiology, Public Health School, Harbin Medical University, Heilongjiang, China; 2 Laboratory of Human Genetics, Beijing Hypertension League Institute, Beijing, China; 3 The First Affiliated Hospital, Medical College of Shantou University, Shantou, China; 4 Fuwai Hospital, Chinese Academy of Medical Sciences, Beijing, China

**Keywords:** Association study, Hypertension, Inflammatory gene, Ischemic stroke

## Abstract

**Background:**

Inflammatory mechanisms are important in stroke risk, and genetic variations in components of the inflammatory response have been implicated as risk factors for stroke. We tested the inflammatory gene polymorphisms and their association with ischemic stroke in a Chinese Han population.

**Methods:**

A total of 1,124 ischemic stroke cases and 1,163 controls were genotyped with inflammatory panel strips containing 51 selected inflammatory gene polymorphisms from 35 candidate genes. We tested the genotype-stroke association with logistic regression model.

**Results:**

We found two single nucleotide polymorphisms (SNPs) in *CCL11* were associated with ischemic stroke. After adjusting for multiple testing using false discovery rate (FDR) with a 0.20 cut-off point, *CCL11* rs4795895 remained statistically significant. We further stratified the study population by their hypertension status. In the hypertensive group, *CCR2* rs1799864, *CCR5* rs1799987 and *CCL11* rs4795895 were nominally associated with increased risk of stroke. In the non-hypertensive group, *CCL11* rs3744508, *LTC4S* rs730012, *FCER1B* rs569108, *TGFB1* rs1800469, *LTA* rs909253 and *CCL11* rs4795895 were associated with ischemic stroke. After correction for multiple testing, *CCR2* rs1799864 and *CCR5* rs1799987 remained significant in the hypertensive group, and *CCL11* rs3744508, *LTC4S* rs730012, *FCER1B* rs569108, *TGFB1* rs1800469, *LTA* rs909253 remained significant in the non-hypertensive group.

**Conclusions:**

Our results indicate that inflammatory genetic variants are associated with increased risk of ischemic stroke in a Chinese Han population, particularly in non-hypertensive individuals.

## Background

Stroke is one of the most common causes of mortality and a leading cause of adult disability worldwide [1]. In China, stroke is the leading cause of death, and ischemic stroke accounts for two-thirds of all strokes [2]. To date, although the etiology and mechanisms of stroke have not been well understood, it is considered as a complex multifactorial disorder with an interaction between the individual’s genetic background and various environmental factors. Previous studies have demonstrated that high blood pressure, smoking, poor diet, abdominal obesity, and lack of physical activities as five common risk factors associated with 80% of all strokes [3]. However, these conventional stroke risk factors do not fully account for the overall risk of stroke. Growing evidence suggests inflammatory processes play fundamental roles in both the etiology and pathophysiology of ischemic cerebrovascular disease [4]. Inflammatory molecules, as well as single nucleotide polymorphisms (SNPs) of genes encoding inflammatory mediators, contribute to the development and progression of a large number of pathological conditions, including cardiovascular diseases [5]. SNPs of inflammatory genes strongly influence the plasma levels and biological activity of the corresponding proteins, with potentially important clinical implications [6,7]. In this study, we investigated the association between history of ischemic stroke and polymorphisms of genes encoding prototypical inflammatory molecules, such as interleukin 4 (*IL-4*), interleukin 6 (*IL-6*), intercellular adhesion molecule 1 (*ICAM-1*), E-selectin (*E-sel*), chemokine (C-C motif) ligand 11 (*CCL11*), lymphotoxin α (*LTA*), and so on.

Although hypertension is the most significant risk factor for stroke [3], evidence from animal and human studies has indicated that some genes predisposing to ischemic stroke are independent of blood pressure [8]. In order to better control the confounding effect of blood pressure in ischemic stroke, the case and control subjects were stratified by their hypertension status.

## Materials and methods

### Study participants

The Stroke Hypertension INvestigation IN Genetics (SHINING) study was conducted by Beijing Hypertension League Institute from 1997 to 2000. SHINING study comprised of subjects exclusive to Han ethnicity. A total of 3,119 participants (1,559 stroke cases and 1,560 controls) were recruited from 6 geographical regions in northern China. Cases were recruited from the community of those who were discharged from hospitals between 1997 to 2000; any stroke patients who suffered a stroke within the past 5 years were eligible to participate the study. All patients had to have medical records with diagnosis from brain computed tomography (CT)/MRI. Control subjects were selected according to the case–control study criteria during the same period (control subjects matched to cases by sex, age within 3 years, geographic location, and blood pressure category (<140/90, ≥140/90 and ≤180/105, >180/105 mmHg)). Details of the protocol have been described elsewhere [9]. Cases with a prior history of myocardial infarction as well as controls with a previous history of myocardial infarction or stroke were excluded.

In this study, we focused only on ischemic stroke (1,124 ischemic stroke cases and 1,163 controls) since it is the most prevalent form of stroke that accounts for more than three-quarters of all cases. Data collected included age, sex, body mass index (BMI), systolic blood pressure (SBP), diastolic blood pressure (DBP) and hypertension. Blood pressures were either measured (one to three measurements) or extracted from medical records. Hypertension was defined as systolic blood pressure ≥140 mmHg or diastolic blood pressure ≥90 mmHg or using of antihypertensive medication.

All participants had signed written informed consent and the study was approved by the ethics committee of Beijing Hypertension League Institute.

### Selection of SNPs and genotyping

DNA was extracted from the whole blood with salting out procedure. We used a polymerase chain reaction (PCR)-based inflammatory marker panel (Roche Molecular Biochemicals, Basel, Switzerland) to genotype DNA samples. Basic information on the selected SNPs is shown in Additional file 1: Table S1. These SNPs are not in linkage disequilibrium. The linkage disequilibrium calculation was performed using Haploview 4.1 software. The SNPs on the strip system represent a selection of genetic variants, essentially all of which had been associated with inflammatory diseases before the development of the strips. The detailed genotyping procedure was described previously [9]. In brief, multilocus PCR was carried out in a single tube containing 51 pairs of biotinylated primers. Each amplified PCR product was hybridized with sequence-specific oligoprobes immobilized on a nylon membrane strip; biotin-based color was developed and captured with a scanner and proprietary software developed by Roche Molecular Systems. Genotyping calls were made by two researchers to yield unanimous results. The call rates of SNPs were more than 98%.

### Statistical analysis

Continuous variables were expressed as mean ± SD and differences between case and control groups were analyzed by Student’s *t* test. Categorical variables were presented as percentage. The *χ*^2^ or exact test was used to identify significant departure from Hardy-Weinberg equilibrium. Polymorphisms with a minor allele frequency (MAF) <5% would be excluded from the analyses.

Logistic regression models were used to estimate odds ratios (ORs) and their 95 percent confidence intervals (95% CIs) by using an additive genetic model. An additive model was built that assumed that the risk for carriers of the heterozygous genotype for developing the outcome was half way between carriers of the homozygous genotypes. The advantage was that the strength of genotype-phenotype association was expressed in a single parameter (best estimate) and statistical tests had only 1 degree of freedom [10]. Also this model would have sufficient power to capture dominant modes of transmission given our sample size. In a first step, age-adjusted and sex-adjusted models were constructed. Thereafter, we fitted multivariable models adjusting for age, sex, BMI and hypertension status. Given the similarity of the results, only multivariable adjusted models are presented in this manuscript. To adjust for multiple hypothesis testing, the false discovery rate (FDR) was applied [11]. The FDR significance threshold was defined with a value of 0.20 [12], meaning that one should expect at most 20% of declared discoveries to be false. A multivariate analysis was performed to find interactions between polymorphisms and hypertension status, adjusting for age, sex, BMI. All analyses were performed using SAS V.9.1.3 (SAS, Cary, NC, USA).

Power calculations were generated using a log-additive model of risk, 5% type 1 error rate. For a minor allele frequency of 0.05, the power in the discovery phase of total group was 86.46%; the power in the hypertension group was 65.50%; and in non-hypertension the power was 57.34%. Power calculations were performed using Quanto software (http://hydra.usc.edu/gxe/).

## Results

The baseline and clinical characteristics of study participants are presented in Table 1. There were 1,124 ischemic stroke cases and 1,163 controls in this study. The control group was older than the case group. There were more individuals with hypertension in the control group and BMI was slightly higher compared to the case group.

**Table 1:** Characteristics of study participants

	**Ischemic stroke patients**	**Controls**	***P *value**
Number of subjects	1,124	1,163	
Age, mean (SD), years	59.20 (10.71)	62.32 (10.68)	<0.0001
Male, %	59.82	61.25	0.483
BMI, mean (SD), kg/m^2^	24.42 (3.00)	25.04 (3.28)	<0.0001
Hypertension, %	64.74	70.19	0.005

Data presented as percentage of participants unless otherwise stated. *P* values for *χ*^2^ test for categorical variables, and Student’s *t* test for continuous variables. Hypertension: systolic blood pressure ≥140 mmHg and/or diastolic blood pressure ≥90 mmHg, or current treatment with antihypertensive medication.

BMI = body mass index.

Among the 51 polymorphisms, 11 SNPs had a MAF <5%. Two SNPs (interleukin 4 (*IL4*) rs2243250 (*P* = 0.001) and nitric oxide synthase 3 (endothelial cell) (*NOS3*) rs1800779 (*P* = 0.012)) deviated from Hardy-Weinberg equilibrium (HWE) in the control group and were excluded from subsequent analysis. Thus, 38 SNPs were selected for further analysis (see Additional file 1: Table S1).

As shown in Table 2, two SNPs in *CCL11* were associated with ischemic stroke (*P* <0.05) in total study population. The ORs (95% CI; *P* value) were 1.47 (1.13 -1.92; 0.004) for *CCL11* rs4795895 and 1.20 (1.01-1.43; 0.042) for *CCL11* rs3744508. After adjustment for multiple testing, *CCL11* rs4795895 remained significant (FDR = 0.152).

**Table 2:** Association between single nucleotide polymorphisms (SNPs) and ischemic stroke

**Gene**	**rsID**	**Minor allele**	**Risk allele**	**MAF, control:case**	**Additive model**			**FDR**
					**OR**	**95% CI**	***P* value**	
*CCL11*	rs4795895	A	G	0.05:0.07	1.47	1.13 - 1.92	0.004	0.152
*CCL11*	rs3744508	A	A	0.14:0.12	1.2	1.01 -1.43	0.042	0.547
*CCR5*	rs1799987	A	A	0.46:0.43	1.13	1.00 - 1.27	0.051	0.547
*TGFB1*	rs1800469	C	C	0.48:0.49	0.9	0.80 - 1.11	0.071	0.547
*NOS3*	rs1799983	T	G	0.10:0.11	1.19	0.99 - 1.45	0.072	0.547
*CCR2*	rs1799864	A	A	0.27:0.25	1.11	0.97 - 1.27	0.143	0.614
*IL6*	rs1800796	G	G	0.33:0.31	1.1	0.97 - 1.25	0.144	0.614
*IL4R*	rs1801275	G	G	0.17:0.16	1.12	0.96 - 1.32	0.162	0.614
*ADRB2*	rs1042713	G	G	0.44:0.42	1.09	0.96 - 1.23	0.172	0.614
*CD14*	rs2569190	T	T	0.37:0.38	1.09	0.96 - 1.23	0.187	0.614
*TNF*	rs361525	A	A	0.05:0.05	1.2	0.90 - 1.61	0.208	0.614
*FCERB1*	rs569108	G	G	0.17:0.16	1.11	0.94 - 1.30	0.217	0.614
*LTA*	rs909253	G	G	0.42:0.41	1.08	0.95 - 1.22	0.227	0.614
*IL1A*	rs1800587	T	C	0.09:0.10	1.12	0.92 - 1.37	0.258	0.614
*LTC4S*	rs730012	C	C	0.15:0.14	1.1	0.93 - 1.30	0.263	0.614
*ADRB2*	rs1042714	G	G	0.07:0.11	1.11	0.92 - 1.34	0.267	0.614
*VDR*	rs2228570	T	T	0.45:0.43	1.15	0.89 - 1.49	0.282	0.614
*IL10*	rs1800872	C	A	0.35:0.36	1.07	0.95 - 1.21	0.291	0.614
*GC*	rs7041	G	G	0.27:0.26	1.07	0.94 - 1.23	0.321	0.642
*VDR*	rs1544410	A	A	0.06:0.05	1.05	0.94 - 1.19	0.387	0.735
*IL5RA*	rs2290608	A	A	0.24:0.23	1.06	0.92 - 1.22	0.435	0.787
*IL1B*	rs16944	T	T	0.49:0.47	1.05	0.93 - 1.18	0.456	0.788
*TNF*	rs1800629	A	A	0.06:0.06	1.09	0.86 - 1.39	0.478	0.79
*CTLA4*	rs5742909	T	C	0.13:0.14	1.06	0.89 - 1.26	0.528	0.81
*CSF2*	rs25882	T	C	0.38:0.39	1.04	0.92 - 1.17	0.533	0.81
*TCF7*	rs244656	T	A	0.12:0.13	1.04	0.87 - 1.25	0.649	0.856
*IL4R*	rs1805015	C	C	0.08:0.08	1.05	0.84 - 1.30	0.669	0.856
*SELP*	rs6131	A	A	0.19:0.20	1.03	0.89 - 1.20	0.674	0.856
*IL13*	rs1295686	T	C	0.28:0.29	1.03	0.90 - 1.17	0.679	0.856
*VCAM1*	rs1041163	C	T	0.15:0.16	1.03	0.88 - 1.21	0.687	0.856
*CXCL12*	rs1801157	A	G	0.21:0.22	1.03	0.89 - 1.19	0.698	0.856
*CTLA4*	rs231775	A	G	0.31:0.31	1.02	0.90 - 1.16	0.738	0.876
C5	rs17611	G	G	0.42:0.42	1.02	0.90 - 1.15	0.768	0.884
*IL4R*	rs1805010	G	G	0.48:0.48	1.01	0.90 - 1.14	0.827	0.899
*ICAM1*	rs5491	T	T	0.07:0.06	1.03	0.80 - 1.31	0.828	0.899
*UGB*	rs3741240	A	A	0.43:0.43	1.03	0.89 - 1.13	0.965	0.977
*NOS2A*	rs1137933	T	T	0.13:0.13	1.00	0.84 - 1.19	0.97	0.977
*GC*	rs4588	A	A	0.32:0.32	1.00	0.88 - 1.14	0.977	0.977

OR was calculated using a reference allele that resulted in an OR >1. *P* value and FDR adjusted for age, sex, body mass index and hypertension status in additive model of inheritance.

FDR = false discovery rate; MAF = minor allele frequency.

Hypertension is a major risk factor for stroke. Evidence has shown that stroke patients with or without hypertension exhibited different clinical features and different pathophysiology [13]. In consideration of the potential effect, hypertension stratification was decided on a priori. Odds ratios for the incidence of ischemic stroke are shown in Table 3. After stratification by hypertension and adjustment for age, sex, and BMI, 3 of 38 SNPs showed significant association (P <0.05) with ischemic stroke in the hypertensive group. These SNPs were in chemokine (C-C motif) receptor 2 (*CCR2*), chemokine (C-C motif) receptor 5 (*CCR5*) and *CCL11*. The ORs (95% CI; *P* value) were 1.26 (1.06 - 1.49; 0.009) for *CCR2* rs1799864, 1.23 (1.06 - 1.42; 0.007) for *CCR5* rs1799987 and 1.45 (1.05 - 1.99; 0.023) for *CCL11* rs4795895. The FDR values were 0.152, 0.152 and 0.291, respectively.

**Table 3:** **Single nucleotide polymorphisms (SNPs) achieving*P*<0.05 for association with ischemic stroke based on hypertension-stratified population**

**Gene**	**rsID**	**Minor allele**	**Risk allele**	**MAF, control:case**	**OR**	**95% CI**	***P*value**	**FDR**
Non-hypertension								
*CCL11*	rs4795895	A	G	0.07:0.05	1.57	1.05 - 2.66	0.032	0.202
*CCL11*	rs3744508	A	A	0.10:0.16	1.64	1.20 - 2.25	0.002	0.076
*LTA*	rs909253	G	G	0.39:0.45	1.29	1.03 - 1.60	0.024	0.182
*LTC4S*	rs730012	C	C	0.11:0.16	1.55	1.13 - 2.12	0.007	0.086
*TGFB1*	rs1800469	C	C	0.46:0.46	1.30	1.07 - 1.59	0.009	0.086
*FCERB1*	rs569108	G	G	0.12:0.17	1.49	1.11 - 2.00	0.009	0.086
Hypertension								
*CCL11*	rs4795895	A	G	0.07:0.05	1.45	1.05 - 1.99	0.023	0.291
*CCR2*	rs1799864	A	A	0.23:0.28	1.26	1.06 - 1.49	0.008	0.152
*CCR5*	rs1799987	A	A	0.43:0.48	1.23	1.06 - 1.42	0.007	0.152

Non-hypertension: cases = 397 controls = 347; hypertension: cases = 727 controls = 816. OR was calculated using a reference allele that resulted in an OR >1. *P* value and FDR adjusted for age, sex and body mass index in additive model of inheritance.

FDR = false discovery rate; MAF = minor allele frequency.

In the non-hypertensive group, after adjustment for age, sex and BMI, there were six SNPs associated with stroke. The ORs (95% CI; *P* value) of these SNPs were 1.64 (1.20 - 2.24; 0.002) for *CCL11* rs3744508, 1.56 (1.14 - 2.14; 0.006) for leukotriene C4 synthase (*LTC4S*) rs730012, 1.48 (1.02 - 2.00; 0.009) for FcepsilonRI-β (*FCER1B*) rs569108, 1.28 (1.03 - 1.59; 0.028) for lymphotoxin α (*LTA*) rs909253, 1.30 (1.07 - 1.59; 0.009) for transforming growth factor β1 (*TGFB1*) rs1800469 and 1.67 (1.05 - 2.66; 0.032) for *CCL11* rs4795895. After correction for multiple testing, five SNPs, *CCL11* rs3744508, *LTC4S* rs730012, *FCER1B* rs569108, *TGFB1* rs1800469, *LTA* rs909253, had FDR values less than 0.20 (Table 3). We tested the interaction between hypertension and genetic variants. There was no interaction detected between any genetic variants and hypertension status (data not shown).

## Discussion

In this case–control study, we assessed the relationship of 51 SNPs in 35 genes related to inflammatory response with the risk of ischemic stroke in 1,124 cases and 1,163 controls. In the control group, there were more individuals with hypertension and BMI was slightly higher compared to the case group. This was due to our effort to recruit controls to match the cases with blood pressure in the search for genes predisposing to ischemic stroke with and without the influence of hypertension.

Initial analysis showed that *CCL11* rs4795895 and *CCL11* rs3744508 were significantly associated with ischemic stroke by adjusting for age, sex, BMI and hypertension status, and after adjusting for multiple testing by FDR, *CCL11* rs4795895 remained significantly associated with ischemic stroke. The *CCL11* rs4795895 genetic polymorphism was the only SNP that retained significant association with ischemic stroke in the whole group. After stratification by hypertension, SNP rs4795895 was still significant in both the non-hypertensive and hypertensive groups, though after adjusting for the multiple hypothesis test it was not significantly associated with ischemic stroke. Polymorphisms rs4795895 and rs3744508 were located in the 5′ flanking region and the coding region of the eotaxin 1 gene, respectively [14]. Previous reports have noted that the presence of the *CCL11* rs3744508 A allele is associated with lower *CCL11* production [15] and an increased risk of myocardial infarction [16]. Ours is the first study to report any association with SNPs of *CCL11* and ischemic stroke. There are two possible mechanisms by which eotaxin might be related to stroke risk. First, it may be the direct consequence of atherosclerosis development. The eotaxin gene and protein are over expressed in human atherosclerosis [17]. Vascular smooth muscle cells (VSMCs) [18] and endothelium [19] in human atheroma prominently express eotaxin, suggesting that eotaxin contributes to the progression of atherosclerosis. Second, increased eotaxin levels can mirror the development of inflammatory responses in heart and brain. Farahi *et al*. reported that circulating eotaxin levels were increased in patients with coronary artery disease [15].

To explore the influence of inflammatory genes on ischemic stroke in the hypertension and non-hypertension groups, we stratified study subjects by their hypertension status and found that *CCR2* rs1799864 and *CCR5* rs1799987 polymorphisms were associated with ischemic stroke in the hypertensive group, and *CCL11* rs3744508, *LTC4S* rs730012, *FCER1B* rs569108, *TGFB1* rs1800469 and *LTA* rs909253 were associated with ischemic stroke in the non-hypertensive group.

*CCR2* rs1799864, a SNP causing an isoleucine-for-valine substitution at position 64 in the CCR2 receptor on chromosome 3, was found to be associated with ischemic cardiomyopathy and myocardial infarction in Czech populations [20,21]. And in an African-American population from the Johns Hopkins Sibling and Family Heart Study, it was reported that the combination of homozygotic or heterozygote *CCR2* rs1799864 genotype and monocyte chemoattractant protein 1 (MCP-1) rs2857656 homozygous genotype was associated with a particularly high prevalence of carotid artery plaque [22]. Chemokine receptor 2 (*CCR2*) is the receptor for MCP-1. Recent studies have indicated that MCP-1 and *CCR2* could have an important role in regulating blood–brain barrier permeability. An animal study had indicated that a lack of *CCR2* greatly reduced brain edema formation and BBB disruption, as well as decreasing leukocyte infiltration, and *CCR2*^−/−^ mice had decreased expression of a wide range of proinflammatory cytokines during reperfusion [23]. Polymorphism *CCR5* rs1799987 is located at position 59,029 in the promoter region of *CCR5* gene. The A allele of the SNP is associated with susceptibility to diabetic nephropathy [24]. Chemokine receptor 5 (*CCR5*) is the receptor for expressed on regulated on activation, normal T cell expressed and secreted (RANTES). RANTES was found as a mediator of cerebral ischemia-reperfusion (I/R) induced blood–brain barrier disruption, tissue injury, and the inflammatory and prothrombogenic phenotype assumed by the cerebral microvasculature after focal I/R [25]. In our study, *CCR2* rs1799864 and *CCR5* rs1799987 were associated with ischemic stroke only in the hypertensive group. We tested the interaction between the polymorphisms and hypertension status, and observed no relationship between them. A possible explanation is that in conditions of high blood pressure, patients carrying risk allele(s) suffer from cerebrovascular accident more than others due to direct or indirect effects of CCR2/CCR5 on blood–brain barrier and blood vessel of brain.

*CCL11CCR2* and *CCR5* belong to the CC family of chemokines. CC chemokines and their receptor polymorphisms have been shown to be associated with a number of infection or inflammation-related disease states, such as atherosclerosis [26]. To the best of our knowledge, our study is the first to demonstrate the association of *CCL11CCR2* and *CCR5* genetic polymorphisms with the risk of ischemic stroke in a Chinese population. CC chemokines may play an important role in both the etiology and pathophysiology of ischemic stroke; the functional relevance of these SNPs to ischemic stroke remains to be further elucidated and CC chemokines could represent promising therapeutic targets in primary and secondary prevention of ischemic stroke.

In our study *TGFB1* rs1800469*, LTC4S* rs730012 and *LTA* rs909253 were associated with ischemic stroke in the non-hypertensive group, which is in line with previous reports [9,27-30], although these findings drew from different ethnic populations and different stroke subtypes. Transforming growth factor β (*TGFB*) is a pleiotropic cytokine with a diversity of effects, such as inducing or facilitating vascular stenosis and thrombogenesis [31,32]. *TGFB1* rs1800469 polymorphism is located in the promoter region and considered possible modulator of expression of TGF-β1 gene and levels. A case–control study had found an increased risk of stroke associated with the risk allele of the rs1800469 in a population from The Netherlands [27]. The rs730012 variant of leukotrieneC4 Synthase (*LTC4S*) upregulates *LTC4S* mRNA expression, increasing the synthesis of proinflammatory leukotrienes [28]. One study found that the *LTC4S* rs730012 variant contributed to large vessel stroke risk in children with sickle cell anemia [29]. Our previous study, a meta-analysis of association of candidate polymorphisms and ischemic stroke in six populations, indicated *LTA* rs909253 was associated with increased risk for ischemic stroke in normotensive subjects but not in hypertensive ones [9]. This is in concordance with the present report.

The β subunit of high affinity IgE receptor (*FCER1B*) is a critical component of the IgE receptor, serving as a chaperone to promote assembly and expression and a strong amplifier of IgE-mediated signals [33]. The *FCER1B* rs569108 G allele was found to have an association with the development of allergic asthma in a Chinese population [30]. In our study, the G allele of rs569108 increased the risk of ischemic stroke in non-hypertensive subjects. To explore the role of *FCER1B* polymorphism in pathogenesis of ischemic stroke, more genetic and functional studies will be needed.

There were five SNPs identified in the non-hypertension group and two SNPs in the hypertension group, however there were many more patients in the hypertension group. One possible explanation is that in the non-hypertensive group, the effect of genetic variation in inflammation gene may be easier to discern. Clinical and experimental studies have reported that hypertension itself is a chronic, low-grade inflammatory process [34]. High blood pressure levels are associated with an increase in circulating levels of inflammation markers that can reflect a vascular inflammatory process [35]. Hypertension could be a stronger factor causing inflammatory process. Therefore, it is possible that in the hypertensive group the genetic influence of inflammatory polymorphisms is concealed. Another possible explanation is that different ischemic stroke subtypes may occur in the different groups stratified by hypertensive status, and stratification could result in a more homogeneous population. According to Arboix *et al*.’s research [13], hypertension was the main cardiovascular risk factor only for lacunar and atherothrombotic infarction. In the present study, different significant SNPs were observed in the hypertensive and non-hypertensive groups, indicating that these polymorphisms may be associated with different subtypes of ischemic stroke, although we could not further define the subtypes. To the best of our knowledge, there is no study with the same design as ours, although hundreds of genetic studies of ischemic stroke have been performed in different geographic and ethnic populations. The ambiguous definition and stratification of ischemic stroke subtypes may be a major reason why no confirmed inflammatory genetic factor was found despite so many research projects on the genetics of ischemic stroke.

There are limitations to our study. First, the current study was a retrospective study, and the included stroke patients were stroke survivors. It could not exclude possible selection bias. Secondly, the cases were not further diagnosed with stroke subtypes, which may confound the results. Thirdly, due to the limitations of the study scope we did not collect information on subjects’ diabetes status, hypercholesterolemia, and coronary artery disease, as well as plasma concentrations of inflammatory markers, which play an important role in the development of ischemic stroke. Also, smoking status was not included in the multivariable analyses. Finally, the results from this study have not been replicated, and should be further validated by a carefully designed study.

## Conclusions

The current study made a survey of inflammatory gene polymorphisms in examining the risk for ischemic stroke. The results were largely concordant with previous knowledge of inflammation and vascular diseases. Our study demonstrated *CCL11* rs4795895 is associated with ischemic stroke after adjusting for multiple testing. We also found five SNPs were associated with ischemic stroke in the non-hypertensive group and two SNPs were associated with ischemic stroke in the hypertensive group. These results collectively showed that inflammatory genetic variants were significantly associated with increased risk of ischemic stroke, particularly in non-hypertensive individuals. Additional studies in larger populations will be needed to confirm the relationship of inflammatory polymorphisms to stroke and its subtypes.

## Competing interests

The authors declare that they have no competing interests.

## Authors’ contributions

NZ, XL, YW carried out the molecular genetic studies and drafted the manuscript. MT participated in the sequence alignment. LY, LM, SW, HZ, LL participated in the design of the study. XL, JL participated in the statistical analysis. XW and JZ designed the study, participated in its design and coordination, and drafted the manuscript. All authors read and approved the final manuscript.

## Supplementary Material

Additional file 1 **Table S1. **Risk allele frequencies, minor allele frequencies and Hardy-Weinberg equilibrium *P* values for the genetic polymorphisms in the Stroke Hypertension INvestigation IN Genetics (SHINING) study. Click here for file
